# Fidelity Assessment of Motion Platform Cueing: Comparison of Driving Behavior under Various Motion Levels

**DOI:** 10.3390/s23125428

**Published:** 2023-06-08

**Authors:** Sara El hamdani, Petr Bouchner, Tereza Kunclova, Přemysl Toman, Josef Svoboda, Stanislav Novotný

**Affiliations:** Department of Vehicle Technology, Faculty of Transportation Sciences, Czech Technical University in Prague, Konviktská 20, 110 00 Prague, Czech Republic

**Keywords:** driving simulator, driving simulator experiment, motion platform, Xsens MTi-G sensor, real car experiment, motion cues fidelity, virtual reality

## Abstract

The present paper focuses on vehicle simulator fidelity, particularly the effect of motion cues intensity on driver performance. The 6-DOF motion platform was used in the experiment; however, we mainly focused on one characteristic of driving behavior. The braking performance of 24 participants in a car simulator was recorded and analyzed. The experiment scenario was composed of acceleration to 120 km/h followed by smooth deceleration to a stop line with prior warning signs at distances of 240, 160, and 80 m to the finish line. To assess the effect of the motion cues, each driver performed the run three times with different motion platform settings–no motion, moderate motion, and maximal possible response and range. The results from the driving simulator were compared with data acquired in an equivalent driving scenario performed in real conditions on a polygon track and taken as reference data. The driving simulator and real car accelerations were recorded using the Xsens MTi-G sensor. The outcomes confirmed the hypothesis that driving with a higher level of motion cues in the driving simulator brought more natural braking behavior of the experimental drivers, better correlated with the real car driving test data, although exceptions were found.

## 1. Introduction

Driving simulator technology is one of the most sophisticated applications of computer-aided kinematic and dynamic simulations [1] designed to simulate the various physical and visual characteristics of driving [2] and widely employed to explore diverse aspects of the transportation landscape [3]. For instance, simulators can play a significant role in testing future innovations such as Autonomous Vehicles (AVs) [4] and evaluating performance as well as passenger experience [5] in different driving scenarios (i.e., road conditions, weather, traffic [6], vulnerable road users [7,8]) which is crucial for the development and validation of these technologies before deploying them in real-world use [9].

Nonetheless, the motion fidelity aspect is a remaining limitation that the driving simulator research field faces [10,11], despite the continuous efforts invested in the enhancement of the motion platform using advanced Artificial Intelligence techniques, including Machine Learning (ML) algorithms [12,13,14]. To tackle this challenge, evaluating the realism and accuracy of existing motion platforms is first necessary. This could theoretically be done in terms of motion in different axes separately (Pitch, Yaw, Roll) or by evaluating specific driving performances such as acceleration (separately in longitudinal motion/combination with lateral motion) to precisely define the remaining limitations compared to realistic motion cues. Previous research used distinct methodologies to assess the fidelity of driving simulators, including subjective and objective evaluations [10]. In addition, particular focus was given to motion sickness as an important parameter evaluating the values of various driving simulator characteristics, among which are vehicle dynamics [15] and motion cues [16,17]. However, few studies have specifically focused on the assessment of the reliability of physical motion cues of simulators.

Berthoz et al. [18] aimed to assess the effects of motion scaling on the driver’s motion perception and genuine driving performance. The study evaluated the subjective preferences of participants for various scaling factors in three cutting-edge simulator systems as a basis for the assessment. This study concluded that driving performance is greatly impaired when motion cues are either very limited or absent, even though the participants preferred scaling factors below 1. A deeper assessment of the effect of motion cues in distinct axes, namely Yaw and Sway Motion Cues, was conducted in the experiments of Lakerveld et al. [19]. Nonetheless, the evaluation concerned the curve driving simulator, which is widely different from existing Six Degrees-Of-Freedom (6-DOF) motion platforms and hence could not reflect this latter’s performance. Salisbury et al. [20] focused on studying the motion cues in high-performance vehicle simulators such as race-car to understand the race drivers’ cueing needs. Thus, the authors designed adapted cueing filters that were devised to provide the driver with the necessary cues to control the car at its maximum performance level. Yet, the study was limited to 2 and 3-DOF platforms. Contrarily, the research done by Cleij et al. [21] tackled the assessment of motion incongruence in a sophisticated 8-DOF simulator due to mismatches between unrestricted visual motion and restricted inertial motion cues. To avoid the time-invariant ratings of total mismatch yield by traditional assessment methods, the researchers proposed a new evaluation methodology describing physical short-duration cueing errors and consisting of continuous drivers’ rating of motion incongruence during passive driving simulations with consistency, still their method relied entirely on subjective evaluation which is a major limitation of the study. To overcome this weakness, the research conducted previously by El Hamdani et al. [22] applied both objective (i.e., performance in the simulator, eye tracking) and subjective (questionnaire) evaluation methods that showed a positive impact of the feedback given by motion cues on drivers’ braking performance and visual focus on the track. However, the latter paper and the previous surveyed works did not compare the level of the motion platform’s cues with those generated by real cars on the roads.

Earlier research [23,24] assessed the simulator fidelity compared to a real car by evaluating driver performance in both environments. However, these studies had various limitations, such as a limited number of participants (i.e., one in a real car vs. six in a simulator) [24], and relied on old methodologies, namely primitive eye tracking [24] and subjective ranking [23]. A recent paper by Loreto et al. [25] aimed to tackle the whiplash problem by recording and comparing dynamic ranges of motion of the passengers’ heads made in a simulator versus a real car, although the real car part was left as a perspective. Xsens MTi-G sensor [26] is a sensor that has been extensively used in recent state-of-the-art experiments for various measurements of, e.g., vehicle acceleration and motion [27,28,29], simulators with motion platforms [30], mobile robots [31,32,33], pedestrians [34,35], and autonomous vehicles [36], and has proven to be useful and reliable in different use cases.

In this paper, we aim to assess the motion cues provided by a 6-DOF motion platform, focusing on longitudinal motion, compared to a real car recorded using the Xsens MTi-G sensor. We evaluate the deceleration performance of 24 participants while accomplishing smooth braking from high speed in both virtual and real environments. Based on the outcomes, we expect to explore some of the limitations of the motion platform and intend to obtain open directions to improve the overall simulator fidelity.

## 2. Materials and Methods

### 2.1. Research Design

The objective of the current study is to investigate how the simulator’s motion cues impact driving behavior, specifically concerning achieving a smooth braking experience towards a designated point. Therefore, we aim to study driving behavior to assess the simulator fidelity by adjusting the motion platform parameters to various levels of motion cues and comparing the outputs with the performance from a real car test, which serves as a reference.

### 2.2. Participants

A total of 24 drivers participated in the virtual reality experiment, with an age range of 20 to 65 years (average of 29.83 years and a standard deviation of 11.30 years). Out of the 24 participants, 5 were females and 19 were males. They were recruited from various backgrounds, including employees, students from CTU in Prague, and volunteers from non-academic fields. The recruitment process was conducted through the university’s email service. The participants’ driving experience (requirement of having a valid driver’s license of at least category B) ranged from 1 to 48 years (average of 12.29 years and a standard deviation of 11.24 years). In addition, 58.33% of the participants drove at least four times a week, while the rest drove a few times a month. Of the 24 participants, 54.17% had prior experience with driving simulators. However, one participant had to drop out due to motion sickness; thus, the data of 23 participants were used in the evaluation of the simulator data.

To obtain real car behavior, two members of our laboratory (an average of 31 years, one female and one male, both active drivers with driver’s licenses) volunteered to replicate the virtual experiment on a real track as flawlessly as feasible in repeated trials. Five records of their drives were used as a reference for comparison and evaluation of virtual driving measurements, as these sensor data showed the most accurate noise-free sensing in the runs.

### 2.3. Apparatus

#### 2.3.1. Driving Simulator

**Figure 1:**
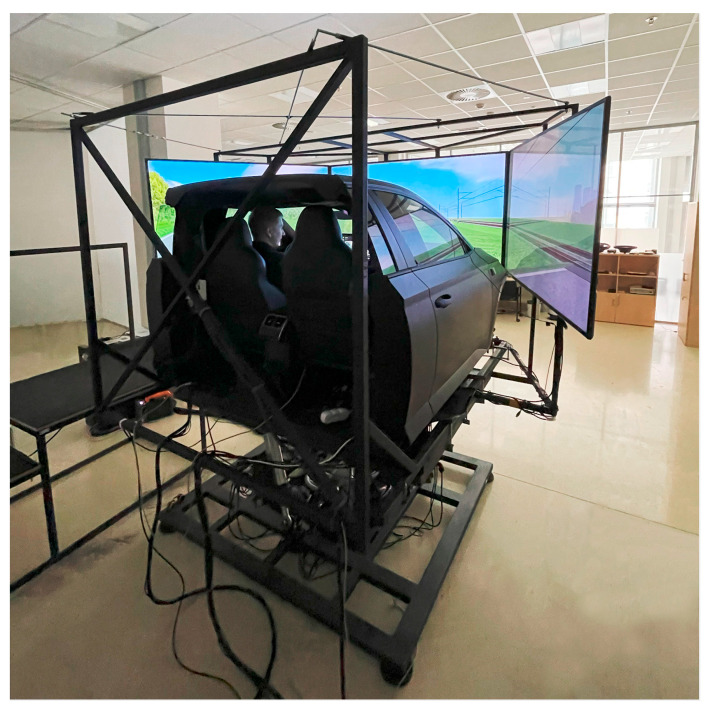
The driving simulator used for the present study in R&D 4.0 LAB at the CIIRC, CTU in Prague.

A laboratory simulator (shown in Figure 1) consisting of a Škoda Superb III cabin with automatic transmission was used for the experiment. The dynamics model [37] of the car corresponded to a front-wheel drive European mid-class car and was developed in the Automotive R&D 4.0 LAB at the Czech Institute of Informatics, Robotics and Cybernetics (CIIRC) at the Czech Technical University (CTU) in Prague. The car cabin was placed on a motion platform with the parameters calibrated to match the driving scenario and follow road irregularities, curves, and acceleration [38]. To provide a fully immersive experience, three Full HD displays were installed to fully cover the entire visible area from the front and side windows and project the scenario. These displays were mounted to the structure of the cabin and thus copied the simulator’s motion. The software part of the simulator incorporated a virtual reality (VR) engine to render the scenario and environment using 3D graphics and spatial audio based on the physics engine.

#### 2.3.2. Motion Platform

**Figure 2:**
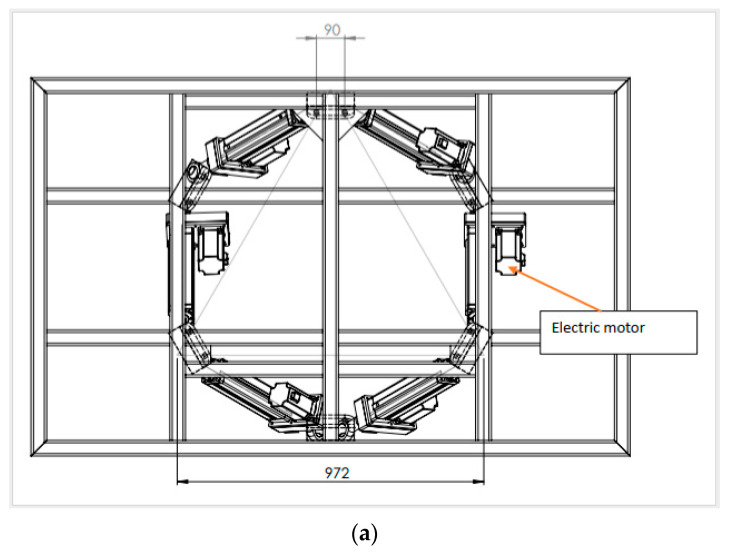

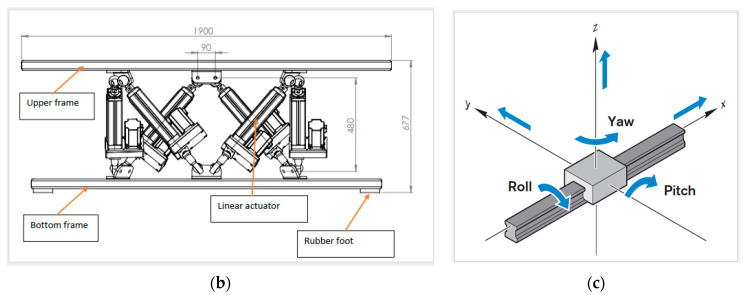
Design of the six degrees freedom motion platform of the used simulator: (**a**) the components of the platform from the top; (**b**) the components of the platform from the side; (**c**) illustrates the Pitch, Roll, Yaw axis enabling 6-DOF for the platform movement.

Figure 2a showcases the motion platform assembly utilized in the laboratory, which is comprised of six electric motors and six actuators located between the upper and bottom frames. With a height of approximately 0.8 m (see Figure 2b), this hardware setup allows movement in the pitch, roll, and yaw axes, as illustrated in Figure 2c, granting the simulator six degrees of freedom (6-DOF). The platform is supplied by Pragolet, s.r.o., Praha-východ, Czech Republic [39] and its movements are managed by a specially designed algorithm for optimal motion cues. This algorithm includes a washout filter and a kinematic transformation of the actuators’ position. The targeted motion and “washout” algorithm are computed on the base of the inner model running on dedicated hardware (PLC). A mathematical and physical model of the vehicle is calculated by separate software on different hardware supplying the control PLC with these target motion data.

#### 2.3.3. Xsens Sensor

**Figure 3:**
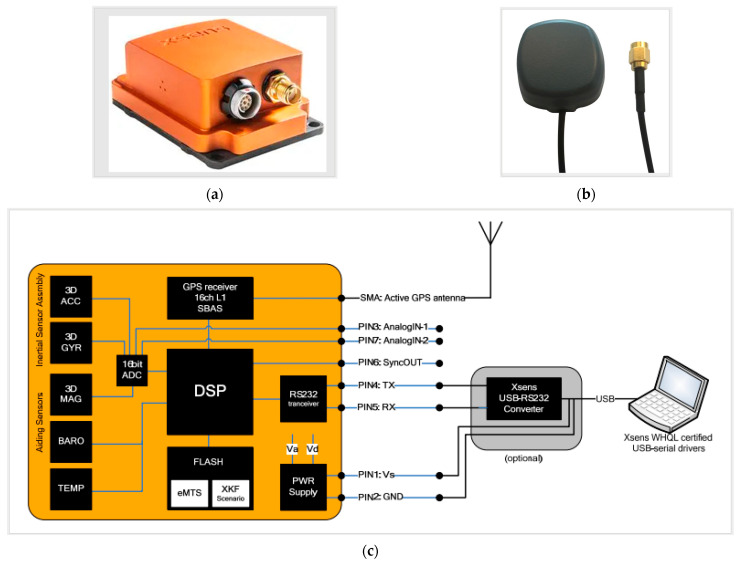
Illustration of the MTi-G sensor used in our experiments: (**a**) sensor “Xsens MTI-G-710-2A8G4”; (**b**) GNSS Antenna; (**c**) architecture overview of the MTi-G system [40].

A sensor by Xsens that provides accurate and reliable motion data in a compact form factor was used to measure the motion-related parameters during the virtual reality and the real track experiments. The Xsens MTI-G-710 sensor (Figure 3a), connected to the GNSS antenna (Figure 3b), is a high-precision motion tracking device designed for use in a variety of applications. The sensor combines GNSS and inertial data and, by using a Kalman filter, provides accurate position and velocity data (with the possibility to work up to 45 s without GNSS data). Based on the architecture illustrated in Figure 3c, this device uses advanced inertial measurement technology to accurately measure and track the movement of an object in three-dimensional space [40].

The MTI-G-710 is equipped with a 6-axis inertial measurement unit (IMU) that consists of a 3-axis accelerometer, a 3-axis gyroscope, and a digital signal processor with the architecture illustrated in Figure 3c and the parameters described in Table 1 [26]. This combination of sensors provides high-resolution motion data with low latency, making it suitable for real-time motion-tracking applications.

Xsens Kalman Filter (XKF-3) computes the orientation of MTi using signals from rate gyroscopes, accelerometers, and magnetometers. It provides a highly accurate 3D orientation estimate with no drift for static and dynamic movements. The XKF-3 algorithm is a sensor fusion algorithm that compensates for drift errors by integrating measurements of gravity from the 3D accelerometers and Earth’s magnetic north from the 3D magnetometers. This drift compensation technique is known as attitude and heading reference, and the system is commonly referred to as an Attitude and Heading Reference System (AHRS) [26].

### 2.4. Settings

#### 2.4.1. Real Track Setup

**Figure 4:**
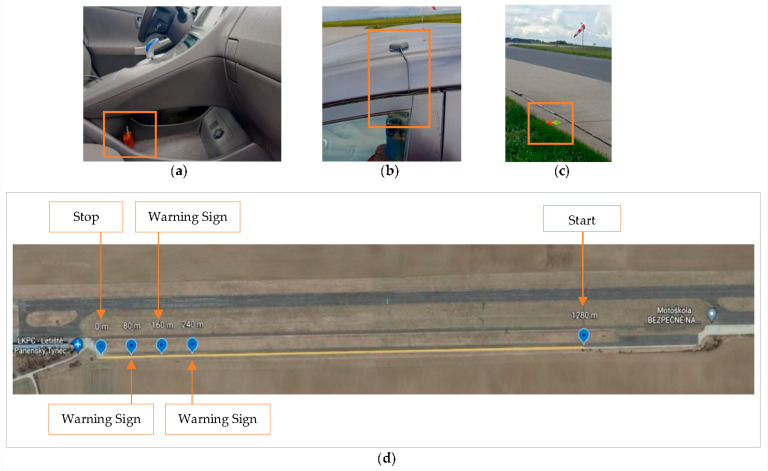
Illustration of the real car experiment setup: (**a**) MTi-G-710 sensor installed close to the center of gravity of a used Toyota car; (**b**) GNSS antenna installed on the top of the vehicle; (**c**) the traffic cones used in the experiment placed on the roadside as a substitute for warning; (**d**) scheme of the real driving experiment track overlaid on Google Earth image.

The real car experiment was conducted to obtain reference data to be used as a typical case with an active driver. In addition, it served us to verify that our motion platform does not operate out of the realistic operational domain. For the experiment, the participants drove the hybrid-powered Toyota Prius vehicle. This car was chosen because of its gearless transmission (e-CVT), which does not introduce any non-linear characteristics into the engine braking. The Xsens MTi-G-710 sensor was installed close to the center of gravity of the car, as shown in Figure 4a. The sensor was connected to the GNSS antenna installed on the top of the vehicle, as presented in Figure 4b. The track consisted of more than 1280 m situated on a safe straight road of an old airport used for vehicular experiments.

The traffic cones, shown in Figure 4c, were placed respectively at the beginning of the track and distances of 240 m, 160 m, and 80 m to the stop line, as were the three warning signs in the simulator experiment, to warn drivers of an impending stopping point and prepare them to initiate deceleration. The positions were depicted on Google Earth in Figure 4d, and this gives the time to gradually reduce the speed to the target from a high initial velocity (≥120 km/h) in compliance with the AASHTO [41] stopping sight distance Equation (1):

(1) $$s=\frac{(0.278\cdot t\cdot v)+v²}{254\cdot (f+G)}$$

where the stopping distance “*S*” is measured in meters, the vehicle’s speed “*v*” is expressed in kilometers per hour, the perception-reaction time “*t*” in seconds with the worst-case scenario being 2.5 s, the road’s grade or slope “*G*” expressed as a decimal, and the coefficient of friction “*f*” between the wheels and the road surface. On a dry road, the coefficient of friction is typically 0.7, while on a wet road, it ranges from 0.3 to 0.4.

#### 2.4.2. Simulation Setup

**Figure 5:**
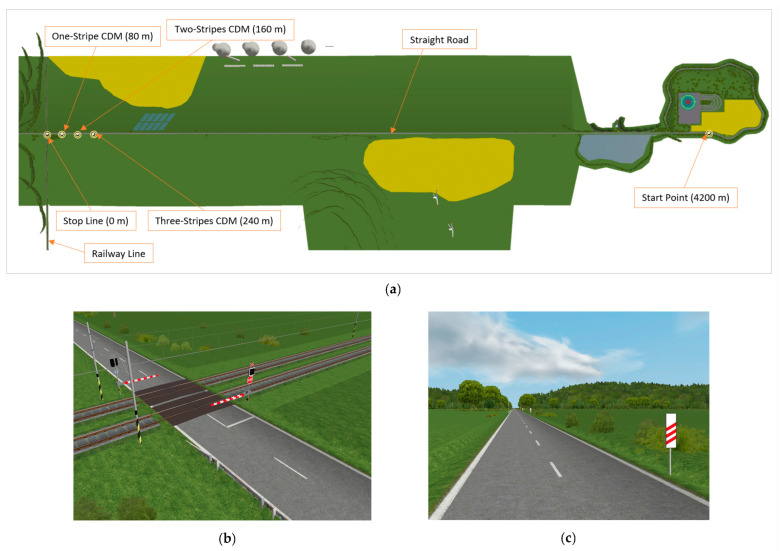
Traffic scenario designed for the virtual reality experiment: (**a**) track layout from the top view with distances to end point; (**b**) the stop line at railway crossing; (**c**) screenshot of the Count-Down Markers (CDM) used as warning signs (80/160/240 m distance).

The driving simulator experiment involved creating a driving scenario on a 4200 m long two-lane rural road with two-way traffic, depicted in Figure 5a. In this scenario, the participants were required to perform a braking task at a specific target point, starting from a 120 km/h driving speed. As shown in Figure 5b, the target stopping point encompasses a railway crossing on the road, and the participants were given instructions to halt directly at the stop line in front of the closed crossing barrier marked with a STOP sign and flashing lights.

The road situation was established by installing warning signs, as shown in Figure 5c, to alert the drivers to the impending railway crossing and prompt them to reduce their speed. As illustrated in Figure 5a, the track includes three unique warning signs with stripes placed at 80 m (three stripes marker), 160 m (two stripes marker), and 240 m (one stripe marker) ahead of the railway crossing, which gives the necessary time to gradually reduce speed to the target from a high initial velocity (≥120 km/h) based on the stopping sight distance in Equation (1).

### 2.5. Procedures

#### 2.5.1. Experiment Procedure

The virtual reality experiment was conducted in a dimly lit and peaceful environment. The participants were initially surveyed for general details like their age or driving experience. A trial scenario was then presented to allow the participants to familiarize themselves with the driving simulator. Novice drivers required an adaptation period of 5 to 10 min, while experienced drivers only took 1 to 3 min to get comfortable.

The participants in the study were instructed to drive a straight path, reach a speed of 120 km/h, and then come to a complete stop at the designated stop line in front of the railway crossing. Each participant repeated the same task three times in each round under different motion levels, namely “Motion Level 0” for no motion cues, “Motion Level 1” for the mild level of motion, and “Motion Level 2” for the high level of motion. The parameters of the motion platform were adjusted to a suitable level of motion before each round based on the measurement plan, meaning that the participants drove under a different order of motion levels.

**Figure 6:**
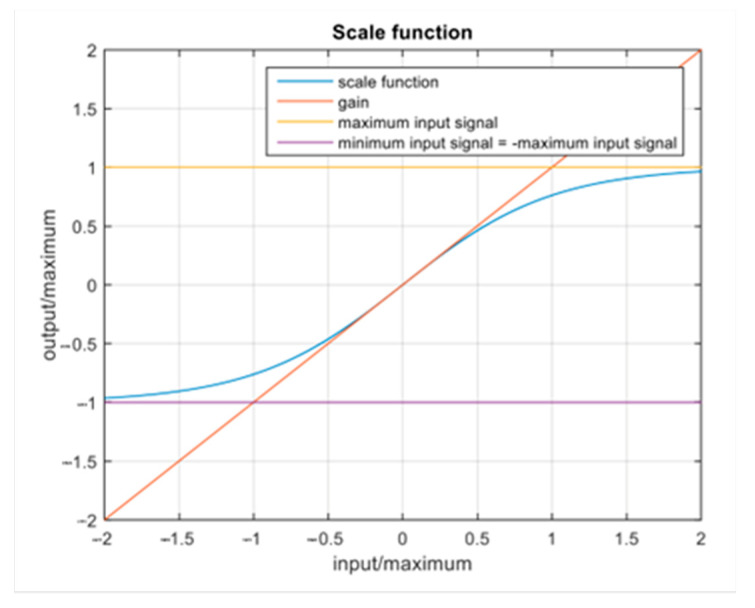
Representation of the Scale Function [25] that scales the input signal from the mathematical model of the simulated vehicle to the motion platform.

The scale function (explained in our previous paper [25]) depicted in Figure 6 scales the input signal from the mathematical model of the simulated vehicle to the motion platform, defined by various parameters including “Maximum Acceleration [rad/s^2^]”, which represents a limit for input linear acceleration, “Maximum Angular Velocity [rad/s]”, which is a limit for input angular velocity, and “Gain [-]”, which is a multiplication factor of linear acceleration. The parameters for the static level were set to 0, as shown in Table 2, where the gain is not taken into consideration. The parameters for the high level of motion were adjusted to the recommended higher values for this simulator to minimize the occurrence of motion sickness. The values for the low level of motion were set by halving the aforementioned values.

On average, each participant’s measurement required 25 min, encompassing adapting to the simulator and completing three rounds of the driving task, each lasting 2.5 min. In contrast, no adaptation time was needed for the real track experiments since the participating drivers from the laboratory had previous experience with the Toyota car selected for the measurements. Volunteers were asked to replicate the virtual braking task as closely as feasible on the real track and were allowed to perform repeated trials.

#### 2.5.2. Analysis Procedure

Two datasets were obtained during the experiment. First, the internal parameters from the physical model of the vehicle simulation were recorded with a periodicity of 2 ms. Second, data from the Xsens accelerometer was collected. The raw data from the driving simulator was filtered and processed so that the individual measurements were referenced to each other based on distance (position). All data were visualized in 2D plots (time × distance; time × acceleration) and checked for missing data/errors. The Xsens data (pitch/acceleration of the platform) were compared to theoretical maximal values. Other recorded parameters, e.g., first brake use prior to the braking task of each driver at different levels of platform motion, were also analyzed. In addition, the experiment survey data were used to obtain the participants’ subjective driving experience.

## 3. Results

The present chapter describes the results obtained from the experiment. Data from the driving simulator and the Xsens accelerometer placed in the simulator during driving were processed. In addition, a comparison with reference data taken from the Xsens accelerometer during real driving testing was carried out.

### 3.1. General

**Figure 7:**
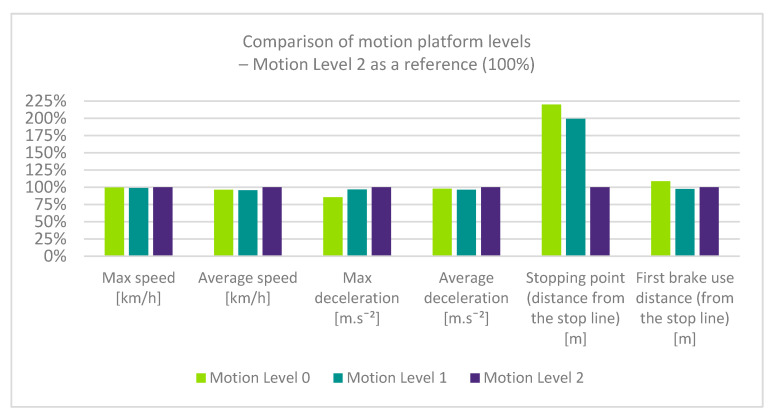
Summary data of driver behavior during the driving simulator experiment (100% reference = the platform cue Motion Level 2).

**Figure 8:**
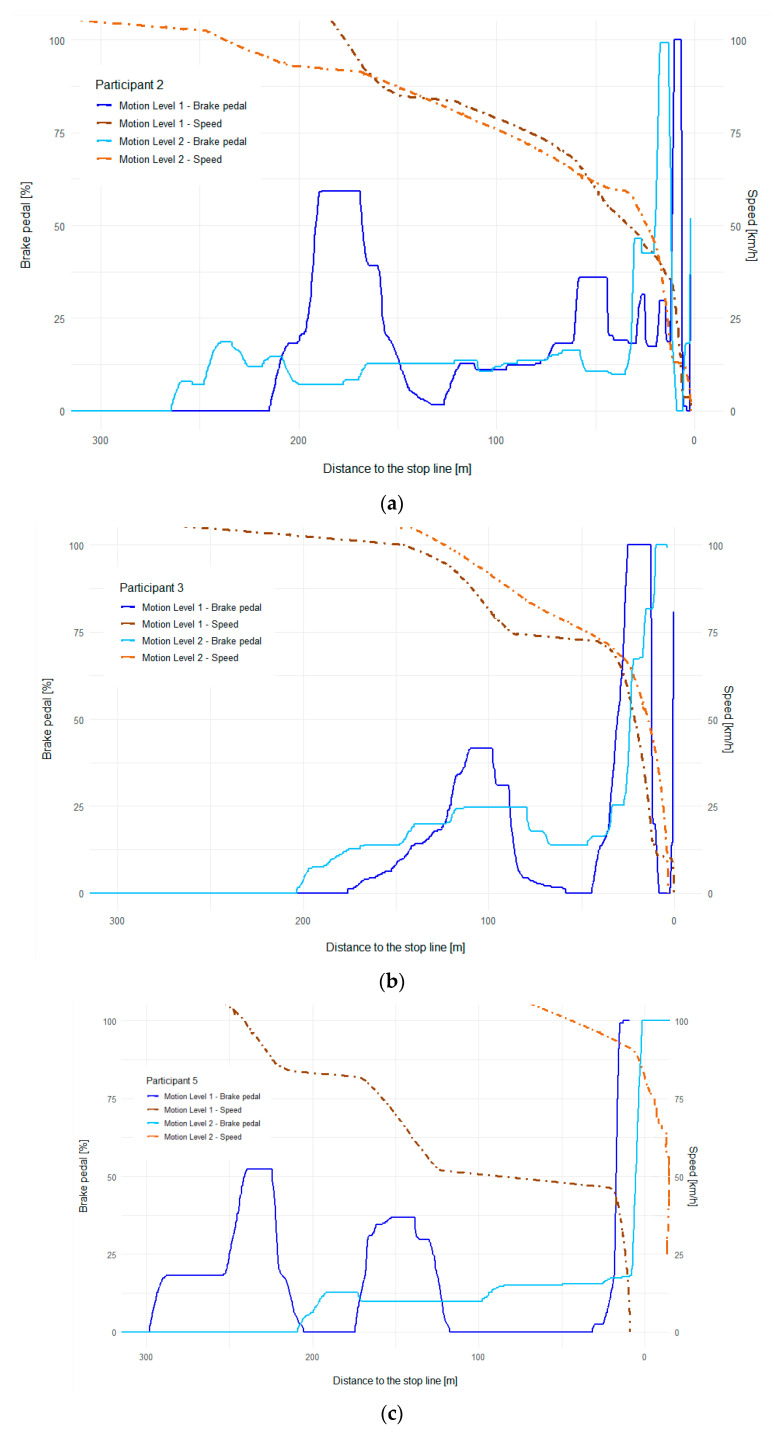
Comparison of braking behavior with Motion Levels 1 and 2: (**a**) braking behavior of Participant 2, (**b**) braking behavior of Participant 3; (**c**) braking behavior of Participant 5.

The summary data shown in Figure 7 indicated very low differences in maximum speed, average speed, and average deceleration when driving with different levels of motion cues. However, other values differed significantly, particularly the accuracy of braking and maximum deceleration. While performing a self-assessment and subjective evaluation of the driving simulator, the test drivers preferred the Motion Level 1 setting, which they considered to be the most realistic and to provide relevant feedback. This fact is confirmed by the summary results, particularly the lowest average speed and the lowest average deceleration. However, a more detailed analysis of braking behavior elaborated in Section 3.2 showed unrealistic aspects of braking with the Motion Level 1 simulator setup. Specifically, we point out a high maximum deceleration and “pumping” of the brake pedal, which would be very uncomfortable in a real vehicle (see Figure 8).

### 3.2. Braking Behaviour

The braking behavior analysis of specific participants in Motion Level 1 and 2 settings provided insight into the perception of deceleration and brake control. The real car driving tests were considered a reference frame of exemplary driver behavior. Based on real car runs, the following assumptions were established: (a) although the drivers had been instructed to brake as continuously as possible, they were unable to do so; therefore, there were distinctive segments with constant deceleration with short transition phases between segments; (b) deceleration greater than approximately 4 m/s^2^ is perceived as too violent to be applied even though it is far from the maximum braking capacity of the vehicle, therefore if such deceleration occurs in the simulator, it is an indicator for an insufficient motion cue.

The braking behavior of various drivers when driving with different levels of motion cues is shown in Figure 8a–c. The driving behavior of all the drivers was analyzed. To present the braking trend, due to clarity reasons, the data of 3 participants were selected for which both trends, for Motion Level 1 as well as for Motion Level 2, are apparent and most clearly demonstrated. These three exemplary trends were selected because they are indicative of and consistent with the driving behavior of the other participants. The decision to present the results in this manner was made because if the trends of all the participants were plotted, the results would become unclear. It can be seen that the participants reacted and started to decelerate earlier under Motion Level 2 conditions, which led to smoother braking and better performance on the braking task.

Additionally, as previously mentioned, the participants were causing so-called “pumping” of the brake pedal under the Motion Level 1 conditions, resulting in abrupt deceleration that would be enormously uncomfortable in a real car. However, these three braking trends are partially different; Motion Level 2 significantly helped Participant 2 feel the vehicle’s speed when braking and therefore began to decelerate earlier and more consistently. While Participant 3 began decelerating later under the Motion Level 2 conditions, and the speed curve trend appears to be steep, this braking was executed more smoothly, without “pumping” the brake pedal as under the Motion Level 1 conditions. Finally, Participant 5 indicated the results of a minority of the participants, with the driver failing to fulfill the task and crossing the given stop line under the Motion Level 2 conditions.

Although he did meet the task under the Motion Level 1 conditions, the braking was not performed smoothly, and again the graph shows “pumping” of the brake pedal that would be uncomfortable in a real car. The participants with similar results can be assumed to have lacked overall control of the vehicle in the simulator and would have needed significantly more adaptation time driving in the simulator than the other participants to complete the task correctly. The question remains how their behavior is reflected in real car driving and whether these characteristics are also evident in real driving, which could then be assessed as unsafe, and the simulator motions would not be the cause of abrupt braking or vice versa.

**Figure 9:**
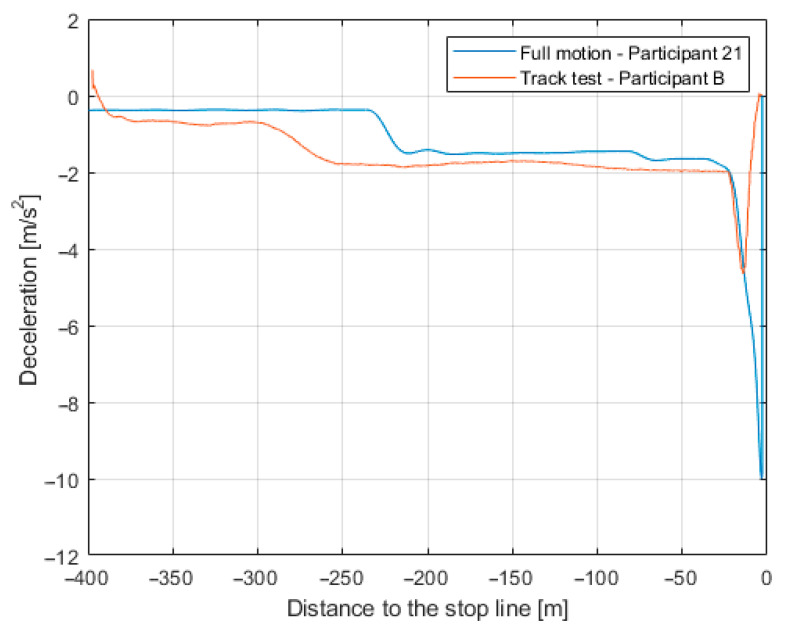
Deceleration in the real driving trial vs. in the driving simulator. Note the difference in peak values.

While the curves of the selected participants’ drives show deceleration peaks (brake pedal press) that are unrealistic compared to real car data of deceleration shown in Figure 9, these peaks are predominantly located in the final braking at low speed (below 5 km/h).

This is typical driver behavior in the simulator caused by a lack of sensitivity in low-speed driving and effort to fulfill the task (stop the car and not let it accelerate again from zero velocity). These peaks are also generally too abrupt to be delivered by the motion platform; hence drivers might not be aware of this short but hard final braking.

In comparison, driving with cues of Motion Level 2 resulted in more stable braking, similar to previously recorded real driving test data. With the cues of Motion Level 1, the perceived deceleration is somewhat limited; therefore, the braking was more erratic.

## 4. Discussion

The results show significant differences in driver behavior corresponding to different motion platform settings when the difference between Motion Level 1 and Motion Level 2 was a maximum of 3° resp. 8°. The Motion Level 2 setting with the highest acceleration and gain parameters correlated with the best driving performance in the driving simulator, i.e., the braking accuracy from the point of view of the final stopping distance from the stop line. However, it is also obvious that even at the maximum settings, the motion platform could not provide motion cues for acceleration/deceleration greater than approximately 2.5 m/s^2^. In comparison to the data from the real car, the significant peaks were observed in maximum deceleration (almost 10 m/s^2^ compared to 4 m/s^2^)–for context, 4 m/s^2^ is perceived as hard braking, while the maximum emergency deceleration of a regular car is 8–9.5 m/s^2^ [42]. The simulator physics engine calculates the movement of the vehicle using an ideal friction coefficient of the tires and tarmac, resulting in a maximum possible deceleration in the vehicle simulator close to 10 m/s^2^.

The present paper broadens the understanding of the relation between motion cues and the quality of driving in a car simulator. The team of authors has previously published a study focused on driver performance and driver eyesight analysis using an eye tracker; however, more data from accelerometers and deeper data analysis are required to understand the effect of different motion cues on drivers in virtual environments. Although the movements of the motion platform are purely synthetic since the 6-DOF platform requires mostly the use of rotation to imitate the acceleration of the vehicle through gravity, this could be perceived well by the driver. However, there are two fundamental limitations of this approach–(a) the necessity of using a washout filter that continuously moves the platform to the default position; (b) the transitory converse effect of rotation into the proper position, which can be perceived as an inverse acceleration to the target one due to rotational acceleration; (c) the capability of this type of motion platform to generate a continuous acceleration/deceleration is physically limited up to 1 G (i.e., approximately 9.8 m/s^2^) in the theoretical case when the platform is tilted fully perpendicular (in reality, however, it is significantly lower, with respect to a feasible tilt around 20°). These limits require a precisely tuned reaction of the motion platform to the requested simulated acceleration. Nevertheless, this study shows that motion cues are beneficial to driver perceptual experience and performance.

## 5. Conclusions

The analysis of the braking behavior in the driving simulator was conducted, focusing on different settings of the motion cues provided by the 6-DOF motion platform. The hypothesis was confirmed that a higher level of motion cues in a driving simulator would yield a closer correlation with real car data. However, unrealistic peak deceleration values were logged. These were explained, but further measurements with optimized parameters of the motion platform could address and resolve this issue. It was also confirmed that the motion platform helps drivers control the vehicle more precisely in terms of specific variables, such as the accuracy of braking to a certain point and smoother braking when required. Thus, it can be concluded that they have a better sense of the vehicle speed in the simulator when the motion platform is used. However, it is necessary to mention and include the exceptions of the drivers whose results indicated that they could not perform the braking task as requested (smoothly to the target) in the simulator, regardless of the motion level. It would be interesting to conduct a real driving experiment with them so that we could consider an adverse effect of the platform or, in case of their better performance in a real car, bring further insights and reasons that could lead to proper optimization of the motion platform parameters.

The motion platform generally delivers a more realistic experience of virtual driving, which is essential for various types of experiments relying on objective measures of driver behavior (e.g., HMI-targeted targeted experiments, driver performance experiments, or even simulator training). On the other hand, the motion cues should be relevant to the driver’s expectations and simultaneously be feasible to perform on a standard (i.e., reasonably priced) motion platform. These requirements naturally remain in opposition to each other, and the designer’s goal is to fine-tune them carefully and based on deep knowledge and experience to provide acceptable and convincing results. Therefore, further studies with the objective of motion optimization would be useful.

## Figures and Tables

**Table 1:** Summary of the main Features and Parameters describing the performance of the MTi-G-710 sensor used in the experiments [26].

Feature	Performance	
	Parameter	Value
Sensor fusion	Roll, Pitch	0.2 deg RMS
	Yaw/Heading	0.8 deg RMS
	Strapdown Integration (SDI)	1.0 m (1σSTD)
	Velocity	0.05 m/s (1σSTD)
Gyroscope	Standard full range	450 deg/s
	In-run bias stability	10 deg/h
	Bandwidth (−3 dB)	415 Hz
	Noise Density	0.01°/s/√Hz
	g-sensitivity (calibr.)	0.003°/s/g
Accelerometer	Standard full range	20 g
	In-run bias stability	15 µg
	Bandwidth (−3 dB)	375 Hz
	Noise Density	60 µg/√Hz

**Table 2:** Summary of the motion platform parameters that define Scale function (Figure 6) tuned for different levels of motion cues.

Motion Level	Maximum Acceleration [rad/s^2^]			Maximum Angular Velocity [rad/s]		Gain [-]		
	*X_Acc_*	*Y_Acc_*	*Z_Acc_*	*X_AV_*	*Y_AV_*	*X_G_*	*Y_G_*	*Z_G_*
Motion Level 0	0	0	0	0	0	-	-	-
Motion Level 1	0.7	0.55	0.7	0.07	0.07	0.45	0.1	0.15
Motion Level 2	1.5	1.1	1.5	0.15	0.15	0.9	0.2	0.3

## Data Availability

Not applicable.
